# Heat Shock Proteins: Stimulators of Innate and Acquired Immunity

**DOI:** 10.1155/2013/461230

**Published:** 2013-05-25

**Authors:** Camilo A. Colaco, Christopher R. Bailey, K. Barry Walker, James Keeble

**Affiliations:** ^1^ImmunoBiology Limited, Babraham Research Campus, Cambridge CB22 3AT, UK; ^2^Aeras, 1405 Research Boulevard, Rockville, MD 20850, USA; ^3^NIBSC, Blanche Lane, South Mimms, Potters Bar EN6 3QG, UK

## Abstract

Adjuvants were reintroduced into modern immunology as the dirty little secret of immunologists by Janeway and thus began the molecular definition of innate immunity. It is now clear that the binding of pathogen-associated molecular patterns (PAMPs) by pattern recognition receptors (PRRs) on antigen presenting cells (APCs) activates the innate immune response and provides the host with a rapid mechanism for detecting infection by pathogens and initiates adaptive immunity. Ironically, in addition to advancing the basic science of immunology, Janeway's revelation on induction of the adaptive system has also spurred an era of rational vaccine design that exploits PRRs. Thus, defined PAMPs that bind to known PRRs are being specifically coupled to antigens to improve their immunogenicity. However, while PAMPs efficiently activate the innate immune response, they do not mediate the capture of antigen that is required to elicit the specific responses of the acquired immune system. Heat shock proteins (HSPs) are molecular chaperones that are found complexed to client polypeptides and have been studied as potential cancer vaccines. In addition to binding PRRs and activating the innate immune response, HSPs have been shown to both induce the maturation of APCs and provide chaperoned polypeptides for specific triggering of the acquired immune response.

## 1. Introduction

The exposure of adjuvants as the immunologist's dirty little secret by Janeway in his seminal introduction to the Cold Spring Harbor Symposium on Quantitative Biology, “Approaching the Asymptote? Evolution and Revolution in Immunology” [1], resulted in a revision of the working model of the immune system and provided a conceptual framework for our current understanding of the innate immune response and its control of adaptive immunity [1, 2]. Janeway reasoned that as the adaptive immune system uses randomly generated receptors to recognise antigen, it cannot reliably distinguish between self and nonself. Adaptive immune cells must thus be instructed as to the origin of an antigen by a system that can determine whether an antigen is derived from self, infectious (i.e., microbial) nonself, or innocuous (i.e., noninfectious and nonmicrobial) nonself. He suggested that the evolutionarily ancient innate immune system might be able to provide such instruction and proposed a mechanism by which the innate immune system could detect an infection and relay its conclusions to the adaptive immune system. Janeway proposed that the innate immune system would detect infection by the use of germ-line encoded pattern recognition receptors (PRRs) to recognise conserved, microbial pathogen-associated molecular patterns (PAMPs). These PAMPs would be unique to microbes and not found in eukaryotic cells so that they would accurately signal infection. Furthermore, they would be common to a broad class of microbes so that a limited number of germ-line encoded receptors could detect all infections and be essential for the life of the microbe so that their detection could not be easily circumvented by mutation. Most importantly, Janeway proposed that the recognition of infection by PRRs on cells of the innate immune system would lead to the induction of signals that resulted in initiation of the adaptive immune response. The subsequent identification of the Toll-like receptors (TLRs) as key PRRs led to an explosion of research on innate immunity and the definition of a number of families of PRRs and signalling pathways that modulate inflammatory responses [2].

Extension of this work into the area of vaccinology has suggested a classification of adjuvants into two major functional groups, those being dependent and independent of binding to TLRs [3, 4]. TLR-dependent adjuvants act directly on dendritic cells (DCs), inducing the upregulation of cytokines, MHC class II costimulatory molecules, and promoting DC migration to the T-cell area of the lymph node [3, 4]. For example, peptidoglycans and other skeletal cell wall components in the Bacillus Calmette-Guérin (BCG) vaccine are recognized by TLR2 and TLR4 and help mediate protective immunity against *Mycobacterium tuberculosis* [5]. Conjugate vaccines against *Haemophilus influenzae* use the outer-membrane proteins from Neisseria to elicit effective adaptive responses via the triggering of TLR2 [6] and the adjuvant properties of short nucleotide sequences containing unmethylated CpG clusters mediated through TLR9 [7]. In contrast, the mechanism of TLR-independent adjuvants like alum and the squalene-based oil-in-water emulsion MF59 remains contentious [2, 8]. Alum has been shown to have immunostimulating activities *in vivo* as it results in the recruitment of monocytes, which take up antigen and migrate to the draining lymph nodes where they differentiate into fully competent inflammatory DCs [8]. Moreover, it has been proposed that adsorption to alum increases antigen availability at injection site, allowing an efficient uptake by antigen-presenting cells (APCs) [8, 9]. However, other studies have shown that alum could also increase antigen uptake by DCs *in vitro* and, in studies on alum as an adjuvant for antigens encapsulated in biopolymers, the improvement in immunogenicity can be correlated to antigen entrapment and release, suggesting that, in addition to the maturation of DCs, alum may also perform an antigen delivery function [9, 10].

Heat shock proteins (HSPs) are ubiquitous chaperones that bind and help fold nascent or denatured polypeptides [11]. HSPs have also been recognised as major immunogens in the immune response against pathogens [12, 13]. These studies, as well as numerous studies on HSPs as cancer vaccines, have revealed that apart from acting as immunogenic antigens themselves, HSPs can also act as adjuvants to stimulate the immunogenicity of heterologous polypeptides to which they are either covalently or noncovalently coupled [13, 14]. Thus it can be argued that HSPs constitute a third functional group of adjuvants. This review will summarise the studies that show that HSPs are not just stimulators of innate immunity but can also traffic antigens into APCs facilitating the induction of specific acquired immune responses. In this context, it is important to note that native HSPs isolated from any organism will carry chaperoned polypeptides that are specific to the source organism and can thus be used directly as vaccine candidates as has been demonstrated by the development of autologous cancer vaccines [14, 15].

## 2. Discovery of the Heat Shock Response and HSPs

The heat shock response was first observed when the temperature of an incubator housing Drosophila was inadvertently elevated, resulting in a change to the pattern of chromosomal puffing within the chromosomes of the salivary glands [17]. Subsequently, a number of proteins were observed to be produced within the same time frame as the appearance of the chromosome puffs and these are what subsequently became known as HSPs [18]. In addition to heat, these proteins were found to be inducible upon exposure to a range of environmental stresses including oxygen deprivation, pH extremes, and nutrient deprivation [19]. This range of responses demonstrated a more general function in providing protection against cellular stress, by limiting protein aggregation and denaturation, and they are thus now more commonly referred to as stress proteins [20]. HSP synthesis occurs at 5–15°C above the optimal environmental temperature of that organism, depending on the organism's growth temperature range [21]. The response is rapid (usually within 2–5 minutes after heat shock), and the expression profile displays a temperature related dynamic, in which the levels of specific HSPs change over the range of different heat shock temperatures [21]. Generally there is a transient increase in the synthesis of HSPs at low level temperature elevation, with a more sustained response observed at higher temperatures, and this pattern of response has been consistently observed in numerous organisms [19–21]. For example, heat shocking BCG at 42°C results in the production of both HSP65 and HSP70, while at 45°C, HSP70 synthesis is more pronounced [22]. At the the transcriptional level, with BCG, the accumulation of mRNA for HSP70 appears to peak at 45 minutes after initiation of temperature elevation, declining after 60 minutes; whereas the elevated mRNA expression of HSP65 mRNA did not persist after temperature elevation to 42°C [22]. The induction of HSPs in mycobacteria can also be induced by other stresses, not the least being phagocytosed by macrophages [23]. The heat shock response and the upregulation of levels of HSPs have been observed in all tissues and in both prokaryotic and eukaryotic organisms, indicating that it is a ubiquitous and critical biological response [21]. The early hypothesis was that these proteins were involved in the stress management of the cells by stabilisation of housekeeping proteins that were critical for survival [20]. Thus, the initial pulsing of cells with low temperature heat stress increased their levels of HSPs and their ability to survive a much higher thermal stress in comparison to untreated cells [21]. However, the more recent demonstration of the constitutive nature of expression of HSPs in all cells strongly suggests that these proteins play a more fundamental role in protein housekeeping within the cell, chaperoning the folding of nascent polypeptides and prevention of protein aggregation [19–21].

Numerous studies have now revealed that HSPs are highly conserved molecules that exhibit a high degree of sequence homology between species [19, 20, 24]. HSPs are found throughout the cell, but different HSP families can be localised to specific cellular locations and can be divided into broad families based on size (see Table 1). The HSPs involved in protein folding can be separated into differing functional systems, with some overlap [20, 24, 25]. The HSP60-HSP10 (GroEL-GroES) system is involved in classical protein folding [24]. The HSP70-HSP40 (DnaK-DnaJ) system stabilises peptides in a linear, unfolded state and delivers them to the HSP60-HSP10 system [25]. Small HSP family members can bind partially folded peptides and mediate their loading onto one of the folding systems (e.g., HSP60-HSP10) [25]. The HSP90 family are found predominantly in the cytoplasm and are thought to mediate the folding of specialised proteins such as steroid receptors and protein kinases [26]. Thermal tolerance, disaggregation, and unfolding of aggregated proteins for enzymatic digestion are handled by the larger HSP100 chaperones [27]. Being involved in such a variety of cellular processes, it is unsurprising that the majority of HSPs (HSP60, HSP70, and HSP90) are fundamental to cell survival, and mutation or deletion of the major HSP genes is often lethal to both cells and organisms [24–27].

The major HSP families are associated with ATPase activity that is essential for their function as molecular chaperones [24, 28]. In the HSP60 system, ATP binding brings about a conformational change that exposes its peptide binding core allowing peptides to enter the peptide binding chamber [28, 29]. This is then followed by the binding of the cochaperone HSP10, which closes the chamber and ATP hydrolysis to ADP and then energises the folding of the nascent polypeptide chain in a hydrophobic environment [29]. In the HSP70 system, ATP binding brings about a conformational change in the HSP that exposes its peptide binding site, allowing peptides to enter the binding cleft and ATP hydrolysis to ADP then closes this cleft [28, 30]. The nascent protein can then undergo folding without interference from other constituents of the intracellular environment [30]. In the HSP90 (Gp96) system, in addition to ADP/ATP, peptide binding is under the control of calcium levels that brings about the required conformational changes for peptide binding [31].

**Figure 1:**
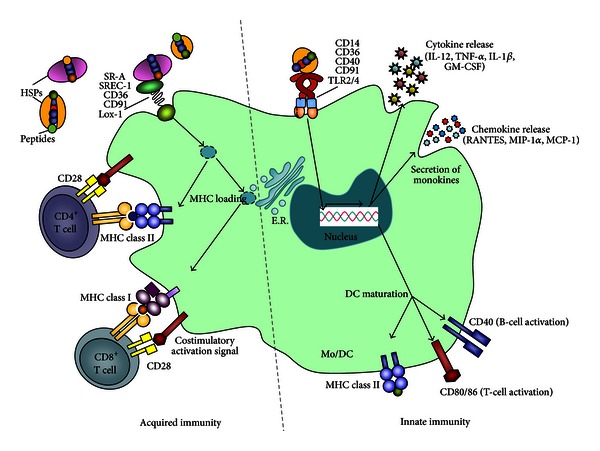
Dual role of HSPs in the activation of both innate immunity, with the induction of monokines and maturation of DCs, and acquired immunity, with the provision of peptides for MHC-loading and antigen specific responses.

While their role as molecular chaperones is their most obvious biological function, their reported functions relating to the immune system are still being elucidated. Numerous studies have implicated HSPs in various aspects of the immunological response to antigens, leading to the proposal that these proteins carry out a “moonlighting” function as “chaperokines” [32–34]. These studies have shown that HSPs act both as adjuvants by triggering TLRs on cells of the innate immune system, in particular macrophages and DCs, and also as carriers of antigens by providing a mechanism for chaperoning polypeptides for the loading of MHC molecules and the subsequent facilitation of induction of acquired immunity [32–36] (Figure 1).

## 3. Innate Immunity

Initially HSPs were thought to be exclusively intracellular proteins that were only released into cellular environment upon cellular injury or necrosis, but not apoptosis and, as such, they were not generally regarded as PAMPs but considered to be “danger associated molecular patterns” (DAMPs) [37]. DAMPs are molecules that serve as alternative ligands for PRRs but signal the presence of cellular damage, as distinct from the presence of pathogens, thus also activating the innate immune response [38]. However it is now apparent that HSPs can be actively secreted into the extracellular environment by tumour cells or released from cells undergoing necrotic lysis in response to cytotoxic lymphocyte (CTL) or natural killer (NK) action, or viral infections [39–41]. Members of HSP60, HSP70, and HSP90 (gp96) families have all been linked with innate immune stimulation [12, 14, 36, 42]. They have been observed to elicit nonspecific cytokine and chemokine secretion from cells of the mammalian innate immune system, to upregulate costimulatory molecules, and to activate APCs in particular DCs via a number of receptors [43–45]. One of the initial HSPs to be studied for its effects on innate immunity was recombinant mycobacterial HSP65 which was shown to stimulate the human monocyte cell line THP-1 resulting in the production of TNF-*α*, IL-6, and IL-8 [46]. In comparison to the mycobacterial HSP65, the mammalian homologue HSP60 was 10–100 times more potent at stimulating human monocytes to secrete cytokines (IL-6, IL-10 TNF-*α*, IL-12, and GM-CSF) [47, 48]. However, despite showing 70% amino acid homology, the two chaperonins appear to mediate the innate immune responses through different cellular receptors [48–50]. For mycobacterial HSP65, signalling occurs primarily via CD14 and can be blocked by the use of antibodies against this receptor: in contrast HSP60 appears to be CD14 independent and may bind and signal via TLR4 [49]. The domains of mycobacterial HSP65 were cloned (apical domain, intermediate domain, and equatorial domain), and the binding to CD14 was localised to the equatorial domain [50].

Mycobacterial HSP70 stimulates cytokine production in monocytes, by interacting with both TLR2 and 4, in a CD14-dependent manner [51]. This ability to activate innate immunity was localised to the C-terminal peptide binding region (aa359–610) of HSP70 which elicited production of IL-12, TNF-*α*, RANTES, and nitric oxide (NO) in THP-1 cells, whereas the N terminal nucleotide binding region of HSP70 (aa 1–358) did not [52, 53]. The full mycobacterial HSP70 molecule appears also to contain epitopes that inhibit DC maturation and promote anti-inflammatory cytokines (IL-10) [54]. Mycobacterial HSP70 can also interact with CD8^+^ T-cells via CD40 to produce RANTES, MIP-1*α*, and MIP-1*β* [55]. Further studies revealed that only mycobacterial HSP70 but not human HSP70 induced this observation, with bacterial HSP70 (DnaK) and human HSP70 appearing to bind to different regions of CD40 on macrophages and DCs [55, 56]. It has also been reported that mycobacterial HSP70 binds to CCR5 and CD40 on human DCs, stimulating production of IL-12p40 and TNF-*α* [57], though this has been contested by other groups that dissociate the innate and acquired functions of both human and mycobacterial HSP70 (aa359–635) [58]. Thus it appears that HSP70's ability to stimulate cells contributing to the innate immune system is dependent on the source of HSP, as mammalian and microbial forms appear to use different receptors [52–58]. Moreover, it appears that while mycobacterial HSP70 stimulates an innate immune response, the situation with mammalian HSP70 is more variable and some members of this gene family may downregulate the immune response instead [34, 44, 54, 59]. This has led to the proposal that these HSPs may have a distinct role as “resolution associated molecular patterns” (RAMPs) that lead to the resolution of inflammation induced by activation of the innate immune response by DAMPs and PAMPs [59, 60].

The eukaryotic family of HSP90/gp96 chaperones has also been shown to interact with TLR2 and -4 and induce the activation of the NF-*κβ* pathway and the subsequent secretion of IL-12 and TNF-*α* [61]. In addition, it has also been reported that gp96 isolated from mouse liver induced the production of NO in both murine (Raw264.7) and human (THP-1) macrophage cell lines and that this action was mediated by the binding of gp96 to CD36 [62, 63]. These studies also report that mixtures of IFN-*γ* lead to a synergistic production of NO from these macrophage cell lines [63].

There is still controversy and conflicting observations about the ability of mammalian HSP to stimulate the mammalian immune system [64]. However there is compelling evidence that bacterial HSPs, including mycobacterial HSP, are able to stimulate the innate immune system with data coming from the study of domains of HSP60 [50] and HSP70 [52, 53]. A complication of initial studies was the copurification of lipopolysaccharide (LPS) as a contaminant in preparations of recombinant HSPs. Thus Gao and Tsan suggested that the biological effect observed with human HSP60 was as a result of LPS contamination as HSP60 with a low endotoxin activity did not result in TNF-*α* production in the murine macrophage Raw 264.7 cell line [65]. However, the use of highly purified HSP60 and the stimulation of innate immune responses by endotoxin-free mycobacterial HSP60 show that LPS contamination does not account for all the observations reported [66]. Moreover, the chemokine stimulatory effects of mycobacterial HSP70 can be blocked by antibodies specific for CD40 but not by inhibitors of LPS [55]. These authors also show that the effect of HSP70 (but not LPS) is lost when digested with proteinase K and the differing responses to different peptide domains of the protein also rule out LPS contamination issues [53–55]. The controversy regarding the potential contamination of HSP preparations with PAMPs has recently been discussed in detail and supports a distinct role for HSPs in the activation of the innate immune response [64].

## 4. Adaptive Immunity of HSPs

The first indication that HSPs could modulate the generation of adaptive immunity derived from observations in cancer studies aimed at elucidating the immunogenicity of sarcomas in genetically identical mice [42, 67]. Biochemical dissection of chemically induced sarcomas identified gp96 as the tumour rejection antigen and cloning of the gene identified it as a member of the HSP90 family [68]. However, immunisation with gp96 elicited sarcoma-specific immunity and gp96 purified from other chemically induced tumours or normal tissue did not elicit immunoprotection although no differences were observed at a protein or genetic level for these HSPs [67, 68]. Srivastava thus proposed that the immunogenicity was conferred by tumour-specific peptides associated with the HSPs and this was supported by the observation that a plethora of peptides could be observed bound to gp96 [42, 68].

Confirmation that immunity was due to the associated peptides was achieved by removal of the chaperoned peptide. HSP70 purified by affinity chromatography on ADP-sepharose retained its chaperoned polypeptides and provided protection against tumour challenge, whereas purification using ATP-sepharose yielded HSP70 that lacked its associated peptides and did not provide protection [42, 69]. HSP70 has a binding pocket that was first demonstrated for the ER HSP70 homolog BiP [70] and later for bacterial HSP70 [71]. The binding pocket interacts with peptides of 8–26 aa in length that are rich in leucine, isoleucine, valine, phenylalanine, and tyrosine [72, 73]. Peptide binding is under the control of ATP/ADP binding to HSP70, which brings about conformational changes that expose its binding pocket [28]. In contrast to HSP70, HSP90 is found as a homodimer and has an open peptide binding cleft that is localised between the long arms of the two monomers [74]. However, like other HSPs, peptide binding is ATP/ADP dependent and HSP90 also functions with cochaperones like HSP40 and HOP [74, 75]. The HSP90 homologue gp96 also contains a binding pocket and, like HSP90, it is an open binding pocket that should allow peptides of any length to interact with it, though the presence of a disulphide bond in this domain may also affect peptide binding [75]. In gp96, peptide binding has also been reported to be under the control of calcium levels that brings about the required conformational changes for peptide binding, a mechanism distinct from other HSP90 homologues [31, 75].

The most interesting feature of the uptake of HSP-chaperoned peptides by APCs is their availability for cross-presentation, which is the ability of exogenous antigens to enter endogenous loading pathway of MHC Class I molecules and thus prime CD8^+^ T cells [76–80]. Cross-presentation can occur via one of two pathways, either the vacuolar/endocytic pathway (nonclassical MHC I loading) or the cytosolic pathway (classical MHC I loading) [78, 79]. In the vacuolar/endocytic pathway, antigen is taken up by the cell by phagocytosis, and formation of phagolysosomes provides the appropriate environment for the production of peptide fragments that are then loaded onto MHC I molecules within this compartment: the source of MHC I molecules is believed to be from membrane recycling or from ER-phagosome fusion [78, 79]. In the cytosolic pathway, antigen is once again taken up by the cell by phagocytosis and, once internalised, the antigen is trafficked to the cellular cytosol (through the transmembrane protein Sec61) and enters the classical MHC I pathway of loading: this translocation to the cytosol requires HSP90 [79, 80].

The cross-presentation of peptides bound to HSPs has been shown to be receptor mediated, with HSP70 and gp96 binding to CD91 and HSP90/gp96 binding to Scavenger receptor-A on APCs [43–45]. HSP70 also binds to Scavenger receptor-A, Scavenger receptor-F1, stabilin-1, LOX-1, and SREC-1 [44, 79]. Although CD91 is found on macrophages, its distribution on DCs is low, suggesting that the scavenger receptors and LOX-1 may be the more common receptors involved in HSP-receptor-mediated cross-presentation [44, 45, 81]. Thus different receptor binding and selective internalisation may account for the enhanced immunogenicity of different HSPs, and upon internalisation, the HSP bound peptide can be taken into the vacuolar/endocytic or cytosolic pathway of cross-presentation. The factors that determine which pathway is taken remain unclear, but it appears to be dependent on both the nature of the bound peptide and the APC cell type [43–45, 76–79].

The ability of mycobacterial HSP70 to cross-present chaperoned peptides onto mammalian APCs has also been investigated [78, 82]. Construction of a fusion protein consisting of mycobacterial HSP70-ovalbumin (OVA) was shown to induce an antigen-specific CD8^+^ T-cell population in vaccinated mice that showed cytotoxic activity against target cells expressing recombinant OVA [82, 83]. Furthermore, Harding and colleagues have shown, *in vivo*, that an extended OVA peptide, noncovalently associated with mycobacterial HSP70, could be presented via the MHC I presentation pathways of bone-marrow-derived murine macrophages and DCs to induce the secretion of IL-2 from a T-cell hybridoma specific for OVA peptide/MHC I complex [78]. This cross-presentation was dependent on the peptide being bound to mycobacterial HSP70 and required active internalisation via CD91 but did not involve interaction with CD40 or TLR [78, 82]. However, treatment of macrophages or B cells with Brefeldin A, an inhibitor of ER to golgi transport and thus the cytosolic pathway of cross-presentation, did not result in a significant reduction of processing and presentation of the fusion peptide, though a significant reduction was seen when DCs were used as APCs [83]. This suggests that, in macrophages and B cells, polypeptides chaperoned by mycobacterial HSP70 are cross-presented predominantly via the vacuolar/endocytic pathway, whereas in DCs cross-presentation occurs via the cytosolic pathway [78, 84]. The ability of mycobacterial HSP to effect cross-presentation has also been observed in human DCs, using an influenza A derived MHC I peptide epitopes fused to various HSP70 domains [34, 84]. These studies showed that, *in vivo*, mycobacterial HSP70 bound peptides were able to cross-present bound peptide, cross-prime CD8^+^ T-cells, and generate CTL that lysed peptide-labeled target cells: surprisingly low quantities of mycobacterial HSP70 peptide complex (120 pM) were required to bring about CTL priming, about 4 orders of magnitude lower concentration than that required to bring about a similar response with unchaperoned peptide [34, 84].

## 5. HSP Cancer Vaccines

The initial work on host-derived HSPs (gp96) from tumours as cancer vaccines has now progressed through preclinical development into clinical trials, generating proof of concept [36, 85–87]. There have been numerous reviews on the preclinical development studies and the reader is referred to these for further details [14, 36, 42]. The most advanced of the clinical trials utilise patient-derived autologous vaccines, called Vitespen/Oncophage, which are gp96 preparations purified from surgically removed samples of the patients' tumours using proprietary methods including affinity chromatography [42, 85]. A range of tumours including metastatic colorectal carcinoma, metastatic melanoma, non-Hodgkin lymphoma, pancreatic adenocarcinoma, and renal cell carcinoma have been studied in clinical trials up to phase III [87]. However, while these vaccines have shown minimal side effects and are well tolerated, their effectiveness as therapeutic agents has been varied [87–89]. In a randomised phase III trial of individuals with renal cell carcinoma, administration of isolated gp96 did not result in a statistically significant improvement in disease outcome [88]. In contrast, when assessed in individuals in stage IV melanoma, individuals that were in substage M1a and M1b (those that had signs of spread to other areas of the skin and lung) did show a delay in disease progression compared to a group that received conventional chemotherapy and/or surgery. However, in the more advanced stages of the disease, no effect was observed [89]. In groups that did show an effect, multiple vaccinations were required (>10), at a dose of 25 *μ*g, and it is thought that the disease stage can apparently modulate efficacy, and also the amount of available tumour tissue available to work with will vary with disease stage [87–89]. One strategy to overcome this limitation involves the use of tumour cells fused to DCs for the purification of larger amounts of HSPs from these fusion hybrids [86]. In animal models, this approach has been shown to yield a more immunogenic vaccine than HSPs purified from tumour cells alone, and this has been ascribed to the improved loading of peptides onto HSPs in the APCs compared to tumour cells [36, 86]. However, it should be noted that the majority of animal studies in oncology use HSP70, not gp96 as in Vitespen, and the autologous peptide binding of the latter is distinct from other HSP90 homologues, both of which may also explain the equivocal clinical results with Vitespen [90]. An alternative strategy that addresses both the issues of HSP heterogeneity and yield is the use of chaperone rich cell lysates (CRCLs) that contain multiple HSPs [91–93]. Cell lysates rich in HSPs, produced by free-solution isoelectric focusing of murine tumour cell lysates, showed significantly improved protection against tumour challenge when compared to the use of single HSPs, and these studies are currently being progressed into human clinical trials [91–93].

## 6. HSPs as Infectious Disease Vaccines

Pathogen-derived HSPs have attracted much interest as potential vaccine candidates against *M. tuberculosis* infection as they have been long recognised as immunodominant antigens in infected individuals [5, 11, 12, 94]. HSP65 is perhaps the most immunodominant in disease models, with an estimated 10–20% of all T cells in infected mice specific for HSP65 [11, 95]. Early investigations showed that recombinant mycobacterial HSP65 could activate murine macrophages *in vivo*, and these cells inhibited the growth of the intracellular pathogen *Listeria monocytogenes*, though they did not induce *in vivo *protection against this pathogen [96]. In TB, early work showed that the macrophage-derived cell line (J774) transformed with a plasmid expressing *M. leprae *HSP65 conferred protection in mice against intravenous challenge with *M. tuberculosis *[96]. Adoptive transfer studies showed that CD4^+^ and CD8^+^ T cells specific to HSP65 were elicited and conferred protection [96–98]. Subsequent work demonstrated that vaccination with nucleic acid (DNA) plasmids encoding the *M. leprae *HSP65 could confer protection in a mouse model, and this approach was extended to other mycobacterial HSPs, including HSP70 [99]. Moreover, mycobacterial HSP65 DNA vaccine was also shown to exert therapeutic action in mice previously infected with TB [100]. However the use of HSP DNA vaccines has become a contentious area as in some postexposure therapeutic studies there appeared to be an exacerbation of pathology [101]. These data have had variable reproducibility, and indeed, more recent investigations have indicated that the poor outcomes may result from a general, rather than specific, stimulation of inflammatory responses induced by DNA vaccines in the TB therapeutic animal model [102].

Improved immunogenicity and protective efficacy have also been observed in animal models using combination vaccination strategies, administering DNA vectors expressing mycobacterial HSPs in conjunction with cytokines or other mycobacterial proteins. Thus, the coadministration of DNA vectors that expressed inflammatory cytokines IL-12 or GM-CSF [103] and the mycobacterial Apa protein [104] resulted in an improvement of the IFN-*γ* recall response in both the mouse and primate models [105]. The therapeutic use of mycobacterial HSP expressing vectors has also been investigated in conjunction with chemotherapy regimens and has been shown to improve the outcome of treatment compared to chemotherapy alone [106].

The use of mycobacterial HSP70 as both an antigen and a component of fusion proteins has also been investigated as potential anti-mycobacterial vaccines. Studies have shown that recombinant BCG that produces soluble mycobacterial HSP70 linked to the major membrane protein II of *M. leprae* activates APCs and cross-prime CD8^+^ cells, resulting in improved protection against *M. leprae* challenge in a mouse model [107]. Induction of a potentially protective phenotype has also been shown by a DNA vaccine construct that expresses mycobacterial HSP70 fused to the secreted mycobacterial protein MPT51: these studies demonstrated that linkage to the 27 kDa C terminus substrate binding domain of HSP70 was apparently sufficient to induce the protective immune response as no protection was observed using the 44 kDa N-terminal nucleotide binding domain [108]. An investigation into the immunogenicity of native and recombinant mycobacterial HSP16 (HSPx) was recently published [109]. This study indicated that native but not recombinant HSP16 (when administered with the adjuvant dioctadecylammonium bromide) could elicit protection in a mouse model of TB, and in addition, it had the capacity to boost an existing BCG vaccination. Although HSP16 has not previously been linked with an ability to chaperone antigenic material and deliver it to the immune system, this study does suggest that native mycobacterial HSPs could exploit this pathway for other mycobacterial components [15, 109].

Finally some investigations have been carried out on the immunogenicity of native (purified) HSP as vaccine candidates toward TB [15, 110, 111]. Host-derived native HSP-peptide complexes from *M. tuberculosis* infected organs have been studied and shown to contain pathogen-derived peptides and, importantly, are capable of eliciting a protective immune response [110]. However, there remain considerable manufacturing and scale-up hurdles to be overcome in this approach for the production of material for large-scale vaccination. In addition, the use of these mammalian, host HSPs as vaccines could have regulatory hurdles through the perceived risk of inducing autoimmunity. An alternative approach for the development of TB vaccines is the use of multiple HSPs isolated from stressed (heat shocked) BCG, and these vaccines have been shown to elicit protective immunity in the mouse aerosol challenge model [111]. The utility of this approach is supported by studies dissecting the immunogenicity of PPD, which show that the HSPs are not just major immunological determinants but are essential for the immunogenicity of other antigens in the mycobacterial extracts [112–114]. As shown in the cancer field, vaccines containing multiple HSP families and associated antigens elicit polyclonal immunity that is more robust than the use of single HSPs. The approach of isolating multiple HSPs from stressed pathogens has been extended to bacterial vaccines where broad strain coverage is an advantage, such as in the development of a meningococcal disease vaccine [115]. Such studies also report novel manufacturing methods and, though several challenges remain, these may present an approach to the development of novel infectious disease vaccines.

## 7. Conclusion

This review has summarised the functional properties of HSPs acting not only as chaperones involved in protein synthesis and degradation but also as the bridge between innate and acquired immune responses (Figure 1). HSPs are therefore natural adjuvants, and their role in vaccine design is currently being exploited in the development of vaccines against cancers and infectious diseases.

## Figures and Tables

**Table 1:** Major prokaryotic and eukaryotic families of HSPs and their characteristics (see [15, 16]).

Hsp family	Structural features	Members	Intracellular location
Small HSPs	Varied, often large oligomeric structures	hsp10, GroES, hsp16, *α*-crystallin, hsp20, hsp25, hsp26, hsp27	Cytosol
HSP40	Dimeric	hsp40, DnaJ, Sis1	Cytosol
HSP47	Monomer Trimer	hsp47	Endoplasmic reticulum (ER)
CCT	hetero-oligomeric complex	TRiC (60 Kd family)	cytosol
Calreticulin	Monomeric	Calreticulin, Calnexin	ER
HSP60	2 stacked heptameric rings	hsp60, hsp65, GroEL	Cytosol Mitochondria
HSP70	Monomeric	hsp71, hsc70 (hsp73), hsp110/SSE, DnaK, SSC1, SSQ1, ECM10, Grp78 (BiP), Grp170	Cytosol Mitochondria ER
HSP90	Noncovalent homodimers	hsc84, hsp86, HTPG, Gp96 (Grp94, endoplasmin)	Cytosol ER
HSP100	Multimeric complexes with hsp70 and hsp25	hsp104, Hsp110, Clp proteins, Hsp78	Cytosol Mitochondria
